# Assessing the role of surface glycans of extracellular vesicles on cellular uptake

**DOI:** 10.1038/s41598-019-48499-1

**Published:** 2019-08-15

**Authors:** Charles Williams, Raquel Pazos, Félix Royo, Esperanza González, Meritxell Roura-Ferrer, Aitor Martinez, Jorge Gamiz, Niels-Christian Reichardt, Juan M Falcón-Pérez

**Affiliations:** 1 Exosomes Laboratory, CIC bioGUNE, CIBERehd, Bizkaia Technology Park, Derio, 48160 Spain; 2 Glycotechnology Laboratory, CIC biomaGUNE, Paseo Miramón 182, 20014 San Sebastián, Spain; 3 Centro de Investigación Biomédica en Red de Enfermedades Hepáticas y Digestivas (CIBERehd), Madrid, Spain; 4 Innoprot SL, Building 502-P1 Bizkaia Technology Park, Derio, 48160 Spain; 5 CIBER-BBN, Paseo Miramón 182, 20014 San Sebastian, Spain; 6 IKERBASQUE Basque Foundation for Science, Bilbao, 48013 Bizkaia Spain

**Keywords:** Glycobiology, Extracellular signalling molecules

## Abstract

Extracellular vesicles (EVs) are important mediators of cell-cell communication in a broad variety of physiological contexts. However, there is ambiguity around the fundamental mechanisms by which these effects are transduced, particularly in relation to their uptake by recipient cells. Multiple modes of cellular entry have been suggested and we have further explored the role of glycans as potential determinants of uptake, using EVs from the murine hepatic cell lines AML12 and MLP29 as independent yet comparable models. Lectin microarray technology was employed to define the surface glycosylation patterns of EVs. Glycosidases PNGase F and neuraminidase which cleave N-glycans and terminal sialic acids, respectively, were used to analyze the relevance of these modifications to EV surface glycans on the uptake of fluorescently labelled EVs by a panel of cells representing a variety of tissues. Flow cytometry revealed an increase in affinity for EVs modified by both glycosidase treatments. High-content screening exhibited a broader range of responses with different cell types preferring different vesicle glycosylation states. We also found differences in vesicle charge after treatment with glycosidases. We conclude that glycans are key players in the tuning of EV uptake, through charge-based effects, direct glycan recognition or both, supporting glycoengineering as a toolkit for therapy development.

## Introduction

Extracellular vesicles (EVs) are well recognised as important mediators of intercellular communication^1^. Able to act across either short or long ranges, EV mechanisms of action are based on the delivery of bioactive metabolite, lipid, protein and nucleic acid cargoes to recipient cells. Roles have been found for EVs in biological processes as diverse as homeostasis, immunity, embryonic development and cancer^2^, whilst clinical use of EVs as therapeutics interventions has shown promise in regenerative medicine and as anti-tumour immunotherapies^3,4^. There are also efforts to take advantage of the natural biocompatibility and systemic delivery potential of EVs by repurposing them as vectors for use in gene therapy^5^ and targeted drug delivery^6^.

Successful translation of EVs will benefit from a complete understanding of EV biology, from synthesis and secretion to cellular uptake and the eventual release of cargo. Some processes have been well defined^7^. The endosomal biogenesis of exosomes, for example, was first observed in 1983^8^ and numerous elements of the molecular machinery involved have since been characterised^9^, to include ESCRT proteins (endosomal sorting complex required for transport)^10^, ALIX (ALG-2-interacting protein X)^11^ and the tetraspanins CD81, CD63 and CD9^12^. ESCRT-independent mechanisms of ceramide-induced membrane curvature have also been reported^9^. Other processes are far less clear, particularly cell recognition of EVs and their subsequent uptake. No universal mechanism of docking to cells has been identified and specific receptor-ligand interactions are likely to differ between EV models, dictated by their specific biomolecular compositions^7,13–15^.

There is a growing appreciation for the role of glycans in EV biology, an otherwise overlooked class of biomolecule in the EV field^16^. Indeed, as of writing the community databases EVpedia and Vesiclepedia make no allowance for glycomics datasets^17,18^. Major findings relate to the capture of exosomes by cells and heparan sulfate proteoglycans (HSPGs) in particular have been implicated. The presence of HSPGs on recipient CHO cells was found to be essential for the uptake of cancer cell-derived exosomes^19^ and similar results have been shown in exosomal communication between hepatic stellate cells^20^. Elsewhere, complementary reports have highlighted the involvement of sialic acid residues. Uptake of exosomes was depressed through interference of sialic acid-binding immunoglobulin-type lectin (siglec) receptors. This effect was observed *in vitro* through antibody and free monosaccharide inhibition of Siglecs in HeLa cell culture^21^ and also *in vivo*, with murine gene knockouts of Siglec-1^22^. Desialylation of EVs has also been shown to alter their biodistribution *in vivo*^23^.

Herein, lectin microarrays were used to analyze surface glycosylation of EVs from two hepatic murine cell lines, AML12 and MLP29. To further explore the role of vesicle surface glycans in EV uptake we also applied glycosidase enzymes to modify the native glycosylation profiles of these two EVs models and then assayed the uptake of the treated and non-modified vesicles by a panel of different cell lines, using flow cytometry and high-content screening. While PNGase F will cleave N-glycans from glycoproteins, the neuraminidase employed in this study removes both α-2,3 and α-2,36 linked sialic acid residues from all EV surface glycans. Across our flow cytometry experiments, we observed an increase in the affinity for EVs after treatment with glycosidases PNGase F and neuraminidase compared with control EVs. High-content screening with 28 cell lines showed that the broader landscape of glycan-mediated uptake is more variable, illustrating a complex code of EV-cell surface recognition. Physical characterization of EVs revealed no significant changes to vesicle size after these enzymatic interventions, suggesting that EV modification with PNGase F and neuraminidase has no adverse effect on EV properties and implying that the observed effects on uptake are determined by the modified glycans. However, vesicle charge was found to be reduced and it is unclear whether the increased uptake is based in either glycan-receptor interactions or is a function of charge and electrostatic effects. Our work contributes to a growing body of studies that bridge EV glycomics and functional studies.

## Materials and Methods

### Cell culture, EV purification and DiI labelling

Laboratory stocks of AML12^24^, MLP29^25^, Sk Hep-1^26^, SH-SY5Y^27^, U2OS^28^, M1^29^ and Huh7^30^ cells were maintained and expanded in DMEM supplemented with 10% (v/v) fetal bovine serum (FBS; GIBCO, Life Technologies), 0.1 mg/ml streptomycin, 0.25 μg/mL amphotericin B and 100 units/ml penicillin (BioWhittaker, Lonza), at 37 °C with 5% of CO_2_.

For EV production, AML12 or MLP29 cells were seeded in 150 cm^2^ Sarstedt tissue culture dishes at 5 million cells per plate and, following a six hour attachment period, were washed with PBS before supplying with 12.5 mL of EV-depleted media (contaminating EVs removed by 16 hours ultracentrifugation at 100,000 × *g*, DMEM without phenol red and containing 25 mM HEPES). Conditioned media was collected after 72 hours and centrifuged at 1500 × *g* for 10 min to remove cellular debris. The supernatant was then passed through 0.22 µm-pore filters and EVs then purified through a standard differential ultracentrifugation method: 10,000 × *g* for 30 min and 100,000 × *g* for 75 min (respective *k* factors of 2103.9 and 210.4). The resulting pellets were resuspended in PBS and pooled where appropriate, before ultracentrifugation at 100,000 × *g* for 75 min. This final EV pellet was resuspended in small volumes of PBS (100–200 µL) and the concentration by protein was determined via Bradford assay using bovine serum albumin (BSA) standards. Aliquots were stored at −80 °C. Ultracentrifugation was performed with a Type 45 Ti rotor (Beckman Coulter, #339160) and polycarbonate bottles (Beckman Coulter, #355622) at 4 °C with maximum acceleration and deceleration.

Pre-labelling of EVs for uptake bioassays was achieved by adding 1 mM Vybrant™ DiI Cell-Labelling Solution (Invitrogen) to the seeding suspension at 2.5uL per 1 million cells, prior to plating. After allowing six hours for attachment and cell labelling, seeding media was discarded and culture dishes washed with PBS to remove excess dye. Plates were then supplied with EV-depleted media and following a further 18 hours of culture the media was exchanged for fresh EV-depleted media to ensure removal of excess DiI and maximize the chance that observed fluorescence derives from cell membrane-containing secretions. Conditioned media was collected after a further 48hrs and processed as above. EV production was typically achieved in batches of 8 culture dishes, totaling 200 mL of conditioned media and averaging 200 million cells with 90% viability at harvest. A control sample of DiI dye background was prepared following the same procedures as indicated above but without using any cells.

### Lectin microarray fabrication

Lectin microarrays were printed as previously reported by our group^31,32^. Printing solutions of 47 different lectins (Supplementary Table S1) were prepared to 0.4–0.5 mg mL^−1^ in printing buffer (PBS containing 1.0 mM D-glucose and 0.01% Cy3 labelled BSA) and 30 µL of each loaded into a 384-well plate along with printing buffer controls. A sciFLEX-ARRAYER S11 noncontact piezo-electric spotter (Scienion, Berlin, Germany) was used to spot these onto an NHS-functionalised glass slide (NEXTERION Slide H, SCHOTT) in six replicates of 0.67nL with a pitch of 0.30 mm, resulting in 14 subarrays of 18 columns by 21 rows. Several PBS blanks were printed to maintain array architecture. Print chamber humidity was maintained at 50% during printing and lectin spotted slides were left to react in the chamber overnight at 18 °C, 75% humidity, before storage at −20 °C.

### Lectin microarray analysis of EVs

Aliquots of native EVs were diluted 1:10 in phosphate buffer (300 mM, pH8.5) and labelled with Alexa Fluor 647 NHS ester (Alexa647; Invitrogen, Thermo Fisher Scientific) at a final concentration of 60 μg/mL, 1 hour room at temperature. Excess dye was then quenched through the addition of an equal volume of 1 M Tris buffer to the volume of dye used. Labelled EVs were aliquoted and treated with either 0.5 U/mL peptide-N-glycosidase F or an α-2,3; α-2,6 and α-2,8-neuraminidase at 0.165 U/mL. These samples plus an aliquot each of untreated EVs and an Alexa647 control were incubated overnight at 37 °C. PNGase F derived from *Flavobacterium meningosepticum* culture was obtained from New England BioLabs whilst neuraminidase of *Clostridium perfringens* origin was purchased from Sigma-Aldrich.

Microarray slides were retrieved from the freezer on the day of use and unreacted NHS groups quenched with 30 mM ethanolamine in a borate buffer for 1 hour before blocking with 0.3 mg/mL BSA in PBS containing 0.3 mM CaCl_2_ and 0.05% Tween-20 for a further hour. Glycosidase-treated EV samples were diluted to approximately 1.6 ng/μL in PBS containing 0.3 mg/mL BSA, 0.1 mM CaCl_2_, 0.1 mM MgCl_2,_ and 0.005% Tween-20, before applying to lectin microarrays for 3 hour incubation at room temperature. Slides were then washed in PBS for 5 minutes, dried by centrifugation and scanned with an Agilent G2565BA microarray scanner (Agilent Technologies, Santa Clara, USA). Fluorescence intensities for all spots were extracted using ProScanArray Express v4.0 software (PerkinElmer, Waltham, USA) and manually normalized to the highest binding signal within each array to allow for comparison between samples. Due to fluorescence saturation for the interaction with DSL this lectin was removed from the data set.

### Western blot analysis

All proteins were detected under non-reducing conditions. Cell extracts were obtained by lysis in 300 mM NaCl, 50 mM Tris pH 7.4, 1% Triton X-100 and protease inhibitors. After clarification by centrifugation at 20 000 g, the supernatant was recovered. The protein concentration of the cell extracts and EVs were determined using Bradford protein assay (Bio-Rad). 5 µg of both cell extract and PBS-resuspended EVs were mixed with NuPAGE LDS Sample Buffer (Invitrogen by Thermo Scientific). The samples were incubated for 5 min at 37 °C, 10 min at 65 °C, and 15 min at 95 °C, centrifuged at 20 000 g for 15 min and supernatant separated on NuPAGE 4–12% pre-casted gels (Invitrogen by Thermo Scientific). Proteins were transferred to a PVDF membrane (Millipore by Merck) that was then blocked for 1 h in 5% milk and 0.05% Tween-20 in PBS. Then, the membrane was incubated overnight at 4 °C with the primary antibody, followed by PBS washing before application of the corresponding secondary HRP-conjugated antibody. Chemiluminescent bands were detected with Pierce™ ECL Plus Western Blotting Substrate (Pierce by Thermo Scientific). Original Western blot images are available in Supplementary Information.

Mouse monoclonal antibodies were purchased from the following vendors: mouse monoclonal antibody against TSG101 (clone 4A10) was obtained from Abcam, against Hsp70 (clone BRM22) was obtained from Santa Cruz Biotechnology, Inc. and against Grp78 (clone 40) was purchased from BD Biosciences. Rat monoclonal antibody against Lamp1 (clone 1D4B) and rabbit polyclonal antibody against LimpII (ab16522) were also obtained from Abcam.

### EV characterisation

Purified EV samples were quantified by protein content using the Bradford method with typical harvests yields from 30 µg to 50 µg. Particle counts and vesicle size data were obtained via Nanoparticle Tracking Analysis (NTA) with a NanoSight LM10 NanoSight system (Malvern Pananalytical, Malvern, UK)^33^. Single samples were analysed in triplicate. Average particle counts per µg of protein of 5.1 × 10^8^ and 3.4 × 10^8^ were recorded for MLP29 EVs and AML12 EVs, respectively. Cryo-electron microscopy (cryo-EM) was performed as previously described by our group^34^.

A Zetasizer Nano ZS platform (Malvern Pananalytical) was employed to analyse the zeta potential of EVs in PBS diluent. The refractive index, viscosity and dielectric constant of PBS were set as 1.334, 0.9236 and 78.5 respectively. EV refractive index was set to 1.375. Data collection was performed at 25 °C with 3 runs of 30 measurements each. For both NTA and zeta potential analysis, 1ug/mL unlabelled EV samples were used.

### EV uptake experiments

A Panel of five human cells lines were seeded into 24-well plates at a concentration of 150,000 cells per well. The panel comprised SkHep-1 hepatic endothelial cells, Huh7 hepatoma cell line, SH-SY5Y neuroblasts, M1 fibroblastoid cells and osteoblast-like U-2 OS cells. After 24 hours of culture, media was exchanged for 250 µL of EV-depleted media spiked with 1 μg of EVs pre-labelled with DiI during cell culture as described above, corresponding to particle doses of 5.1 × 10^8^ EVs from MLP29 and 3.4 × 10^8^ EVs from AML12. EVs with native glycosylation were used as controls alongside those modified by PNGase F or α2-3,6,8-neuraminidase. Glycosidase-treated EVs were prepared as directed above with an additional clean up step using Total Exosome Isolation Reagent (Invitrogen) as per manufacturer instructions.

Following 16 hours incubation of cells in the presence of labelled EVs, culture media was discarded and the wells were washed with PBS before harvesting of cells into 0.3 mL 1X TrypLE Express (Gibco). Flow cytometry was performed with a BD FACSCanto II instrument (BD Biosciences, San Jose, USA) and 45 second acquisitions under a medium flow rate were used for data capture. Data analysis was performed using FlowJo software with gating of cells in the PE channel and no impact was found for equivalent DiI background samples. The population percentage of fluorescence positive cells for each sample was taken and normalised to that of the untreated MLP EVs as an uptake index, allowing for comparison between experiments. Within experiments, quintuplet independent replicates were used for each cell line/EV condition alongside control cells. Moreover, each experiment was performed in duplicate using entirely independent preparations of EVs to account for batch-to-batch variability. A range of EV amounts were trialled during development of the flow cytometry assay and single 1 μg doses found to produce sufficient response. Experiment data are available in Supplementary Dataset S2.

Confocal microscopy was performed on samples generated in the same manner. In place of the TrypLE harvest and subsequent flow cytometry, coverslips of cells were fixed with 2% formaldehyde for 20 minutes and washed in PBS before mounting using ProLong Gold Antifade Reagent with DAPI (Invitrogen). Slides were imaged using an LSM 880 laser scanning confocal microscope (ZEISS, Oberkochen, Germany) with a 20x objective.

### High-content screening of EV uptake

High-content screening was performed by Innoprot, S.L. of Derio, Spain. Cells lines were maintained at 37 °C with 5% of CO_2_ in specific culture media and with vessel coatings as described in Supplementary Table S3. Screening was performed using 96-well Imaging Plates from BD with cells seeded at a density of 15,000 cells/well. Triplicate wells of cells were supplied with 250 ng of labelled EVs or a 500 ng equivalent of DiI background in media volumes of 100uL. Plates were incubated for 16 hours and media were then removed and cells incubated with calcein probe and Hoescht for 20 minutes, following manufacturer protocols, before images were taken using a BD Pathway 855 High-Content automated image platform with an Olympus 20X dry objective.

The changes in the fluorescence patterns of the calcein labelled cells and DiI labelled EVs were processed and quantified using image analysis algorithms with the Attovision bioimaging software (BD) in 3 × 3 subfields of each well (500–1000 cells analyzed per well). The excitation and emission filters used were the following: for Hoescht, 380/10 nm and 450/10 nm; for calcein probe, 488/10 nm and 540/20 nm; and for DiI, 548/20 nm and 570LP. The EVs engulfed by the cells were analysed by calculating the number of red particles (EVs) within the calcein signal (cells) utilizing BD AttoVision 1.6/855 software. For this purpose, images were segmented into regions of interest (ROIs) representing the surface area of each cell employing filters in Hoescht (Shading, Rb75x75, 150 < pixel size) and GFP (Blur a lot, Rb75x75, automatic 1–8) signals. EVs were segmented filtering the DiI (Rb2x2, TopHat7x7, intensity 609–495, 2 < pixel size < 50). Resulting values were normalized to the fold change in fluorescence compared to the DiI background and termed uptake index. Experiment data are available in Supplementary Dataset S4.

## Results

### Lectin microarray analysis of native EV glycosylation

**Figure 1:**
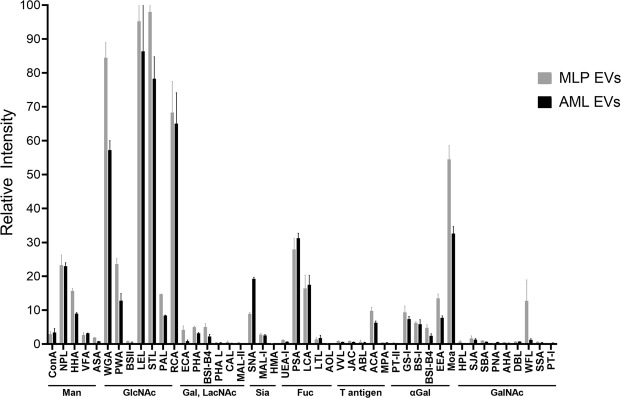
Lectin array analysis of native EVs from MLP29 and AML12. Fluorescence for individual lectin spots was normalised to the highest signal for each samples before combining six lectin spot replicates for average values and standard deviations.

Intact EVs purified from AML12 and MLP29 cell cultures were fluorescently labelled with Alexa Fluor 647 and incubated with microarrays comprising 47 lectins, before washing and fluorescence scanning (Fig. 1). Complex and varied lectin specificities have been simplified to broad saccharide preferences so as to group lectins for easier comparison between samples. Similar lectin binding profiles were observed for both types of vesicles, with equally high fluorescent signals observed for N-acetylglucosamine binding lectins wheat germ agglutinin (WGA), *Phytolacca americana* (PWA) and *Pseudomonas aeruginosa* PA-I (PAL), amongst several other lectins such as *Marasmium oreades* agglutinin (MOA) and *Euonymus europaeus* (EEA). Elsewise, there are clear differences between the EV samples. The presence of fucosylated glycans in both samples can be inferred from strong binding to *Pisum sativum* lectin (PSA) and *Lens culinaris* lectin (LCA), although AML EVs exhibit a higher degree of fucosylation. From the sialic acid binding lectins, AML12 EVs interacted more strongly with *Sambucus nigra* lectin (SNA) than MLP29 EVs, indicating a higher degree of α-2,6 sialylation in the former. Meanwhile, *Maackia amurensis* lectin-I (MAL-I) is specific for α-2,3 linked sialic acid residues and has binding signals that are more consistent between the EV models. A final difference can be observed in the binding of MLP EVs to *Wisteria floribunda* (WFL), suggesting an N-acetylgalactosamine signal that is absent for AML EVs. Certain lectins reached signal saturation and were removed from the analysis to allow for normalization of the data. These were further examples of N-acetylglucosamine binding lectins but also *Narcissus pseudonarcissus* (NPL) that is specific for mannose structures.

Overall, our data shows some agreement with the conserved glycan signature previously described for EVs^35^. Namely, signals corresponding to α-2,6 sialylation, complex type N-glycans and high-mannose N-glycans. However, we also show differing degrees of sialylation between the samples and also a high degree of fucosylation, in line with a recent lectin microarray analysis of EVs from human mesenchymal stem cells^21^. Considering the potential range of glycan variations from different tissue types and organisms, it is perhaps worth updating the concept of a universal glycosylation signature for EVs.

### Glycosidase treatment of EV surface glycans

**Figure 2:**
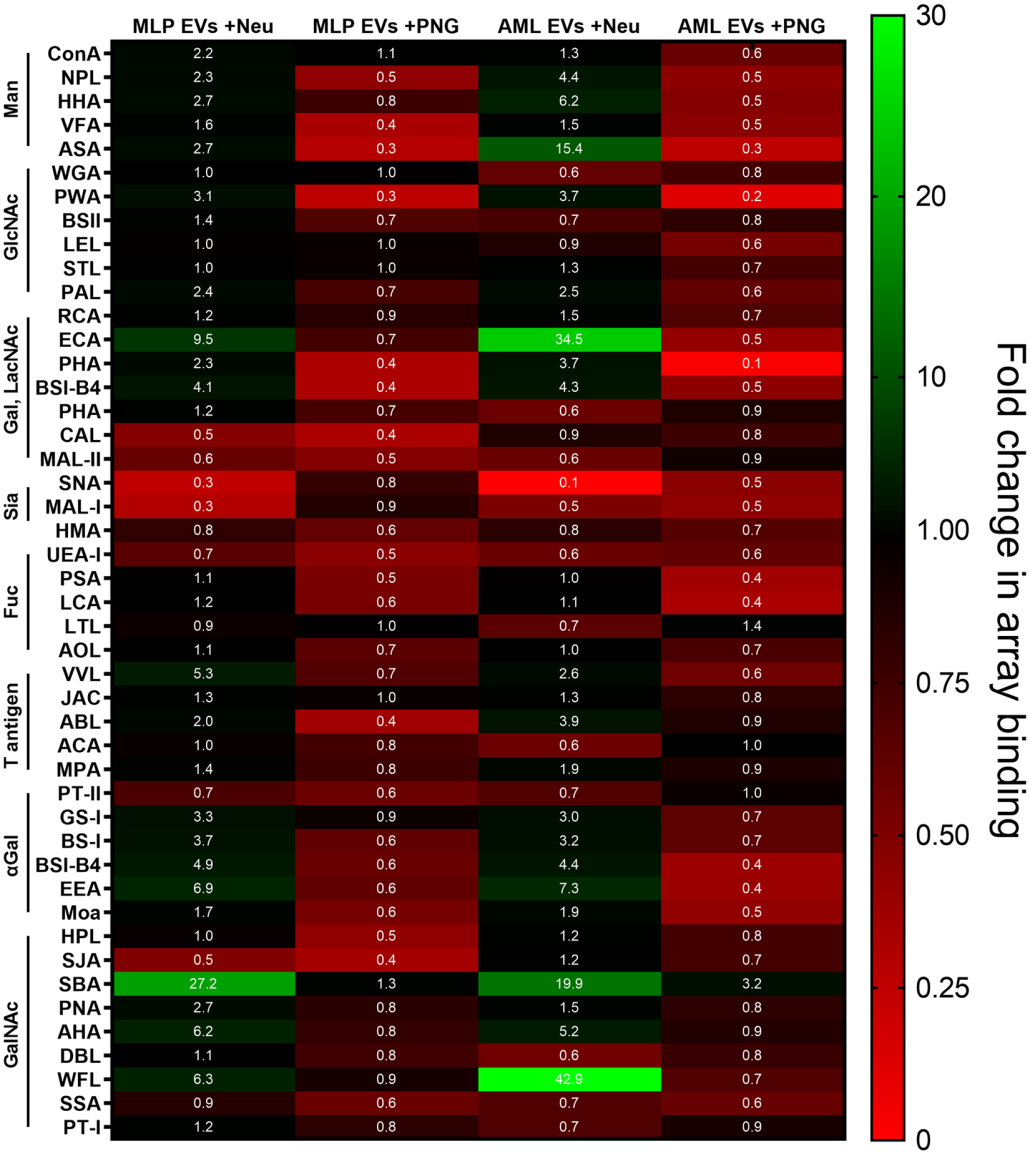
Heat map visualisation for lectin array analysis of EVs after neuraminidase (Neu) or Peptide-N-Glycosidase F (PNG) treatments. The normalised data represent the fold change in fluorescence signal from array binding, relative to EVs of native glycosylation.

In order to modify the EV surface glycosylation we employed the glycosidase enzymes Peptide-N-Glycosidase F, which specifically cleaves N-linked oligosaccharides from the asparagine residues of glycoproteins, and α-2,3- α-2,6- α-2,8 neuraminidase, which trims sialic acid residues from glycolipids, N-glycans and O-glycans. PNGase F was chosen as a maximal intervention as the removal of an entire glycan class would provide a fundamental shift to the presentation of antigens by the EVs in a manner that maintains vesicle integrity. Considering O-glycans and glycolipids, no facile, enzymatic methods currently exist for complete deglycosylation, rather requiring disruptive chemical approaches. Fortunately, N-glycosylation is often the major form of protein glycosylation that impacts protein stability and modulates the accessibility for receptors^36^ and so presents as a useful target. Elsewise, neuraminidase cleavage of terminal sialic acid is comparatively non-invasive on a structural level, although with potentially significant impacts as the enzyme can function on all glycan classes. There is also compelling evidence that sialic acid receptors are involved in exosome uptake^21,22,37^ and also that these residues contribute to the negative charge of EVs^38^. Removal may therefore alter physicochemical attributes of vesicles and affect capture in a receptor-independent manner. These glycosidases are marketed for use with denatured protein samples and so we made some modifications to the digestion protocols, increasing the incubation time from 1 hr to 16 hrs in an attempt to achieve full de-N-glycosylation or desialylation. Efficiency of the treatments was then assessed using lectin microarrays (Fig. 2).

Following neuraminidase treatment, binding to sialic-binding lectins is decreased. This is most notable for the lectin SNA where the signal is nearly completely ablated, whereas changes to MAL-I binding are smaller and less robust. Tellingly, there are corresponding signal increases for galactose and lactosamine binding lectins ECA, RCA and PWA which is consistent with an effective removal of sialic acid revealing underlying galactose residues. Also of note is the decrease in binding to *Lotus tetragonolobus* lectin (LTL), indicating a loss of sialyl-Lewis X epitopes through removal of sialic acid^39^. Moreover, the effect is lessened for MLP29 EVs than for AML12 EVs. This observation provides evidence that the neuraminidase enzyme is acting on O-glycans and implies that EVs from the AML12 cell line have more or more accessible sialyl-Lewis X moieties on their surfaces - a finding that was not evident during direct comparison of the native EVs. Altogether, these data suggest that our chosen reaction conditions for neuraminidase digestion are sufficient for sialic acid removal from intact vesicles.

Interpretation of the glycosylation profiles for EVs subjected to PNGase F digestion is more complex. For both EV models, a decrease in all lectin binding signals was observed, indicating a general decrease of glycosylation in the sample. Indeed, binding to *Phytolacca americana* lectin (PWA) and *Phaseolus vulgaris* agglutinin (PHA) is nearly completely lost compared to the profile of native EVs, although this effect is more pronounced for AML12 EVs than those of MLP29. The strong drop observed in binding to PWA after PNGase F treatment suggests that the majority of terminal β-galactose and lactosamine residues present on the EV surface reside on N-glycans, rather than O-glycans or glycolipids. *Phaseolus vulgaris* agglutinin (PHA, includes both isoforms L and E) binds to a larger binding motif on complex glycans including bisecting GlcNAc. Here, the decrease in binding can be interpreted as a loss of complex N-glycans as they are the only class of glycans that presents these more extended binding motifs^40^. Finally, LTL can again be remarked upon. Sialyl-Lewis X antigens only occur as O-glycans and the fact that no changes are observed is consistent with the mode of action of PNGase F.

Such evidences suggest a successful digestion; however, we cannot tell from our data whether the remaining signals are entirely due to the remaining O-glycans and glycolipids of the samples or whether incomplete de-N-glycosylation has occurred. For both glycosidases, EVs with modified glycosylation profiles were produced but an exact measure of digestion efficiency cannot be made through lectin microarrays alone.

### Western blot analysis

**Figure 3:**
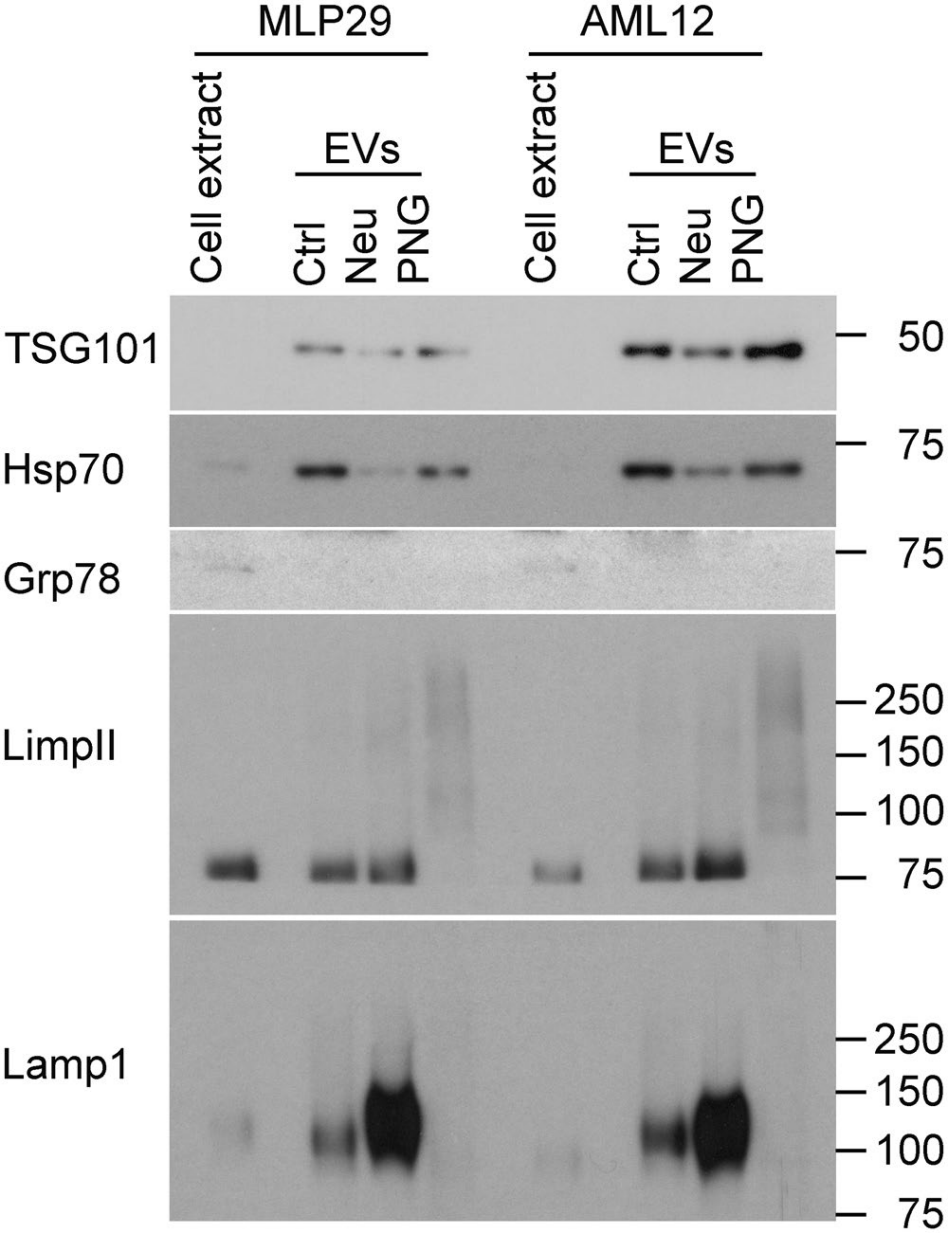
Western blot analysis of native and glycosidase-treated EVs. Cell extracts and EVs were analysed by Western-blotting using antibodies against non-EV (Grp78), EV (Tsg101, Hsp70, LimpII, Lamp1) proteins. Note changes in the detection of glycosylated membrane proteins (LimpII and Lamp1) due to glycosylation treatments. This figure has been composited from a single membrane stained multiple times, with individual images available in Supplementary Information.

Western blot analysis of MLP29 and AML12 EVs showed the enrichment of EV marker proteins TSG101, Hsp70, LimpII and Lamp1, compared with extracts of the producing cells (Fig. 3). Grp78 was included as negative control and showed no detectable contamination of EVs with non-vesicular membranes. Interestingly, the antibody-mediated detection of Lamp1 and LimpII demonstrated sensitivity to glycosidase treatment of EVs, indicating changes to the glycosylation states of these proteins. Treatment of EVs from both cell lines with the neuraminidase enzyme caused a minor decrease in TSG101 and Hsp70 detection but a hugely inflated signal for Lamp1 and a slight increase in LimpII. Alternatively, use of PNGase F resulted in complete ablation of antibody binding in the case of Lamp1 and a far lower staining intensity for LimpII. Gel bands corresponding to LimpII are located further towards the top of the membrane, indicating slower migration which generally correlates with a larger protein size, here an impossibility considering the removal of posttranslational glycan structures. A potential explanation is that the removal of structurally integral glycans results in less constrained protein isoforms of larger size that are unable to travel through the gel. This explanation of a conformational shift would also be sufficient to explain the reduction of detectable antibody binding for Lamp1 and LimpII, in that the epitope is lost after removal of stabilising glycans. An alternative explanation is that the antibody recognises a glycan epitope that is lost after glycosidase treatment. Interpretation of the signal amplification and shift after neuraminidase treatment is more difficult, possibly relating to enhanced accessibility of the binding epitope following desialylation.

Both Lamp1 and LimpII are lysosomal transmembrane proteins with heavily N-glycosylated luminal domains^41^. The presence of these N-glycans has been shown to protect Lamp1 from degradation in the proteolytic environment of the lysosome^42^ but, in the context of exosome biogenesis, the inward budding of the lysosomal membrane to form multivesicular bodies^9^ means that these domains are instead flipped to the extravesicular face. As such, these proteins become accessible for treatment of intact EVs with glycosidases and the stark results from our Western blot analysis are useful in providing further evidence of successful modification to EV glycosylation under our chosen reaction conditions. For researchers wishing to manipulate the glycosylation of EVs but lack the capacity for glycomic analysis, these antibody clones may be tools to verify successful changes to EV glycosylation.

### EV characterisation

**Figure 4:**
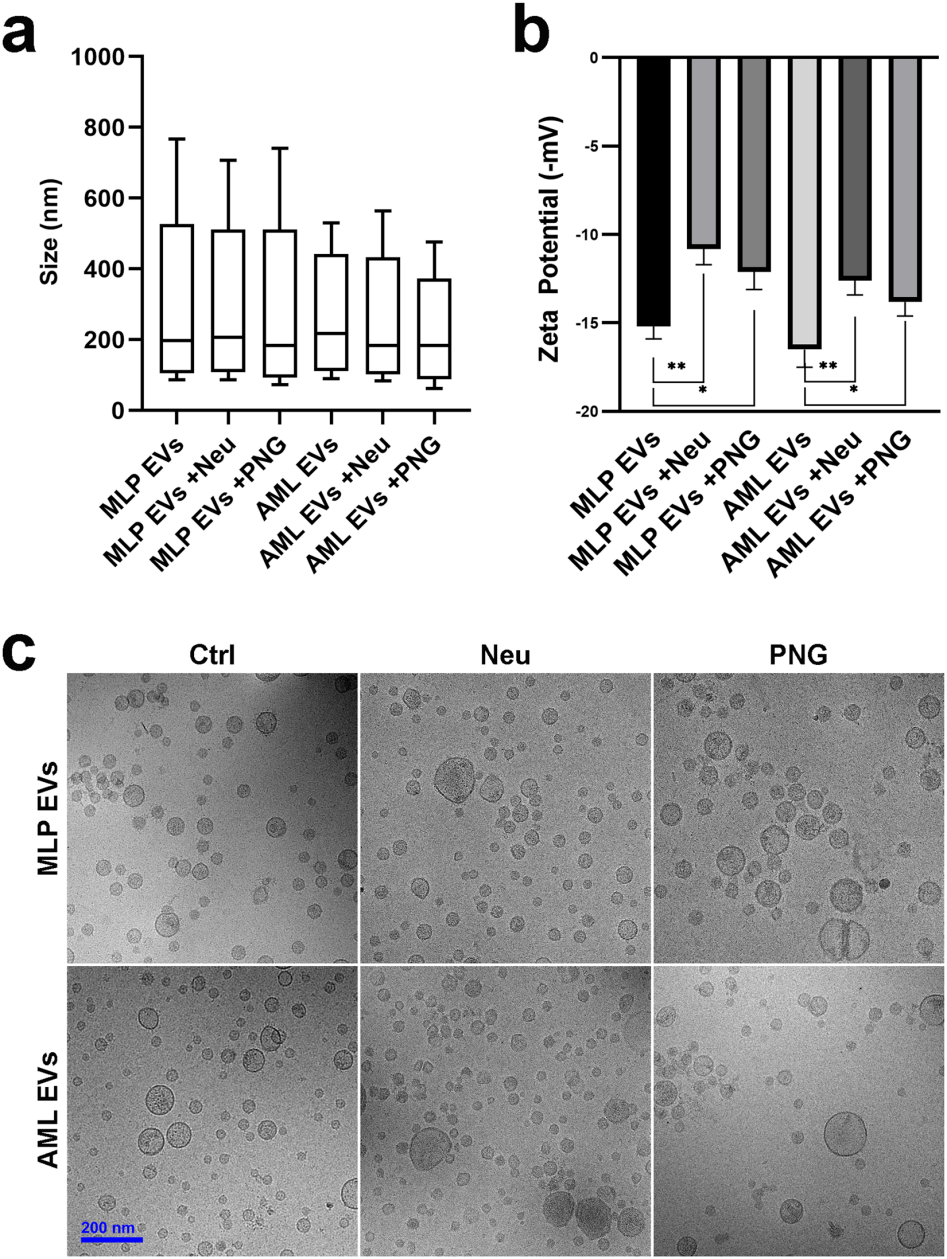
Physical characterisation of EV models. (**a**) Nanoparticle tracking analysis (NTA) of vesicle diameter sizes. Box plots show D10, mean and D90 parameters whilst whiskers exhibit minimum and maximum particle diameters observed. (**b**) Zeta potential determination of particle charges. Significance is calculated by Student’s t test from technical replicates (*p < 0.05, **p < 0.01, error bars represent S.D.). (**c**) Cryo-electron microscopy (cryo-EM) images.

Measurement of vesicle size distributions with NTA revealed no significant changes for EVs after either of the glycosidase treatments (Fig. 4) and cryo-EM images of the glycoengineered EV models corroborated these findings (Fig. 4c). Further physicochemical characterisation was performed by looking at the zeta potential of the EVs – a measure of particle charge^43^. Generally, EVs from the AML12 cell line were more negatively charged than those of MLP29. This is in agreement with the array data, whereby a higher binding signal was observed for SNA by AML12 EVs corresponding to a higher concentration of sialic acid residues. In both EV models, neuraminidase treatment caused a substantial decrease in particle charge from negative to neutral states (Fig. 4b). This effect was also apparent with PNGase F treatment, although less pronounced. Glycan-dependent EV charge can be attributed to the presence of negatively charged HSPGs and sialylated glycans. As such, these shifts in charge reflect our lectin microarrays results, whereby neuraminidase depleted EVs of the majority of sialic acid residues whilst PNGase F was less efficient at doing so due to inactivity with glycolipids and O-glycans. Proteoglycans are the predominant charged glycan species of cell surfaces and so the fact that neuraminidase and PNGase F treatments have measurable effects on EV surface charge is interesting. This may be explained by low concentrations of EV proteoglycans or by reductions in charge caused by HSPG truncation during EV biogenesis^44^.

In summary, glycosidase treatments caused reductions to vesicle charges whilst vesicle sizes and physical appearances did not change significantly, excepting slightly decreased size in the case of AML12 EVs. A known impact of reducing particle charge to more neutral states is aggregation^45^ but these data suggest that this has not occurred in our system. EV aggregates could feasibly skew the results of downstream uptake assays, either through increased fluorescence signals or as a function of increased surface area causing artefactual increases in adhesion to cells and subsequently inflated uptake. As such, we were confident that our assay results reflect the uptake of discrete vesicles.

### EV uptake experiments

**Figure 5:**
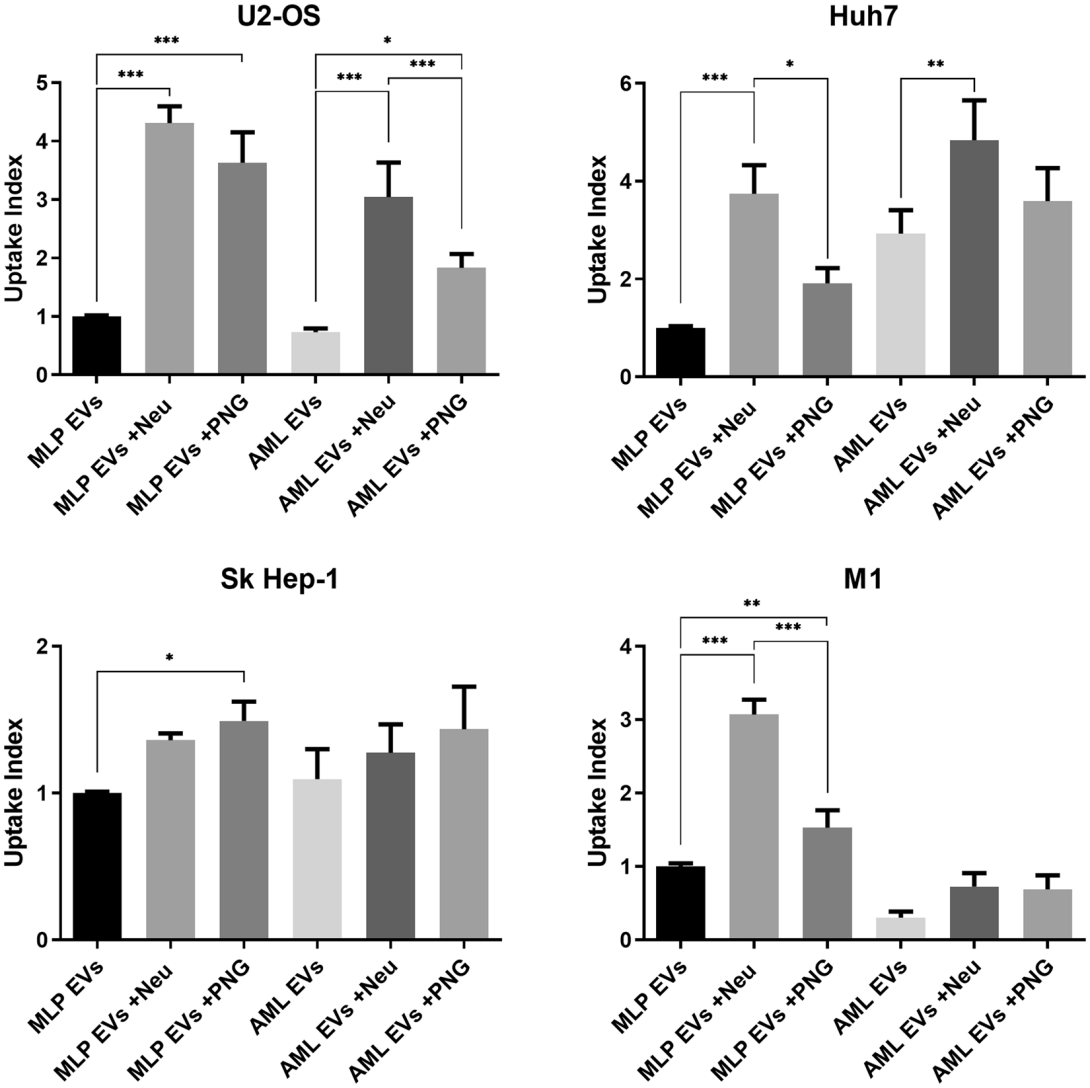
Flow cytometry uptake analysis of glycosidase treated EVs by recipient cells. Uptake index is determined from the percentage of cell populations exhibiting fluorescence after 16 hours, normalised to MLP EVs. Significance is shown relative to native EVs and was calculated by one-way ANOVA, with error bars representing S.E.M. of 10 replicates over two experiments (*p < 0.05, **p < 0.01, ***p < 0.001).

To study the role of EV glycans in cellular uptake, we observed the differences in the uptake of glycoengineered EVs by a panel of four human cell lines. Cell lines were chosen as representative of different organ types and included hepatic cell lines Huh7 and Sk Hep-1, M1 fibroblastoid cells and U2-OS osteoblast-like cells. Incubation of cells with our fluorescent EV models and subsequent, quantitative flow cytometry analyses revealed consistent increases in preference for glycosidase-treated EVs compared with control EVs (Fig. 5). As a general rule, desialylated EVs were preferred by cells over EVs treated by PNGase F, with some exceptions. Notably, this effect is not consistent between the two hepatic cell lines, Sk Hep-1 (endothelial origin) and Huh7 (hepatoma cell line), as Sk Hep-1 cells show a slight preference for PNGase F treated EVs. Moreover, the uptake of all AML EVs by M1 cells was generally lessened to the point where statistical significance cannot be concluded. The magnitude of uptake affinities differed strongly between cell lines and ranged from around 1.5 fold increases in the case of the Sk Hep-1 cells to almost 5 fold for U2-OS and Huh7. Further experiments using equivalent amounts of a DiI background control did not yield detectable fluorescence, indicating validity of these results. From these varied results we can conclude that different cell types will respond to alteration in EV glycan composition in different ways, supporting glycosylation as potential source of targeting ligands for tuning specific cellular sites.

**Figure 6:**
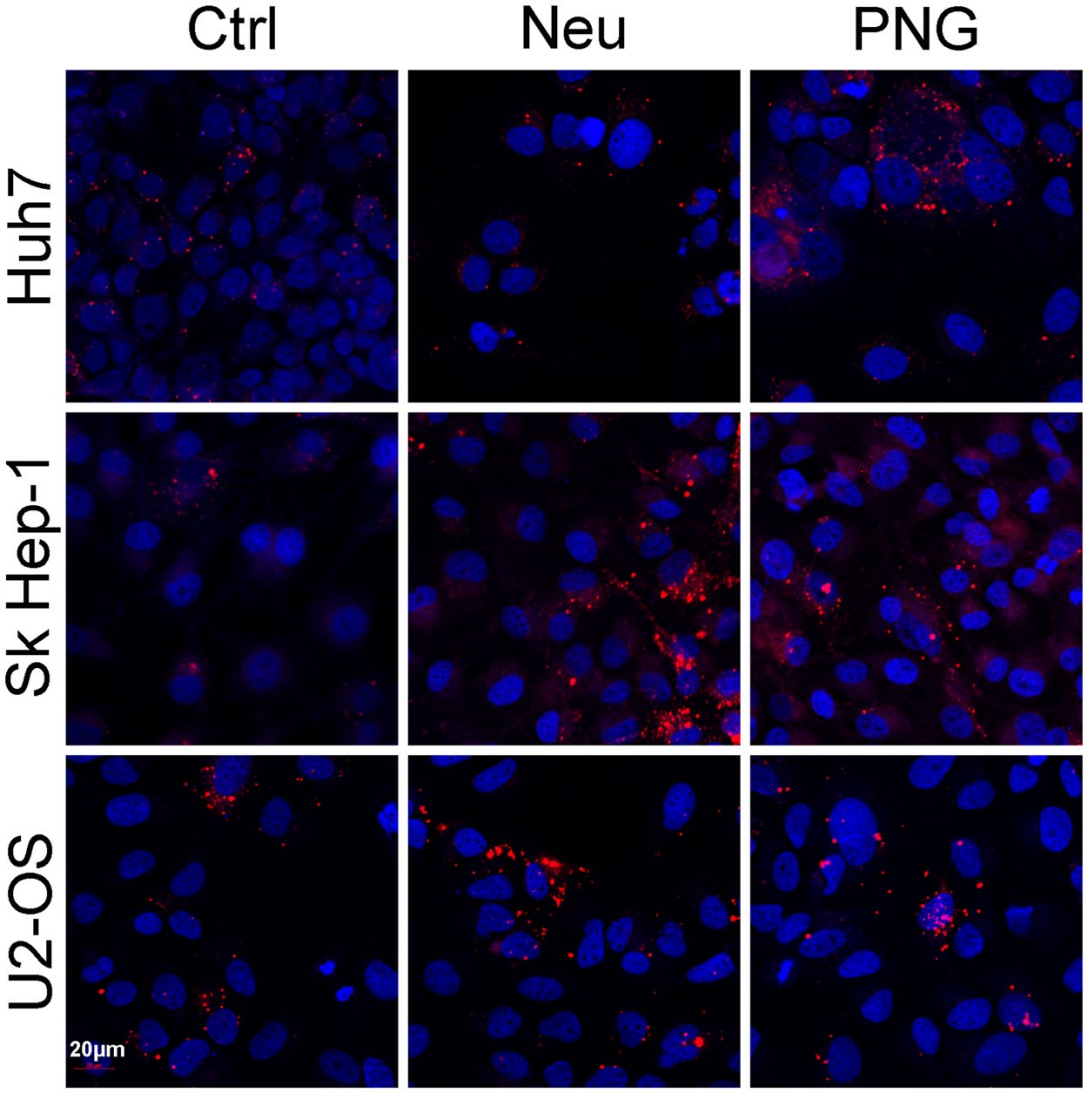
Confocal microscopy uptake analysis of glycosidase-treated AML12 EVs. Cells were incubated in the presence of DiI-labelled AML12 EVs for 16 hours. Confocal images of DiI (red) and DAPI (blue) dyes were taken at 20x magnification (20 µm bar for scale).

To interpret these results, we have used the term ‘uptake’. However, in the case of flow cytometry, there is not the resolution to distinguish between fluorescence of internalized vesicles or from those at the surface. Prior to harvest of cells for flow cytometry, culture vessels were subjected to PBS washes in an attempt to remove any non-specific EVs adhered to the cell surface. To provide a qualitative confirmation that our experimental parameters are looking at *bona fide* uptake we have also employed confocal microscopy (Fig. 6). Huh7, Sk Hep-1 and U2-OS lines were cultured in the presence of DiI-labelled EVs obtained from AML12 cells. From the images it is evident that vesicles are being internalized by cells. In agreement with flow cytometry data, the confocal microscopy images also support the preference for glycosidase-treated EVs.

### High-content screening of EV uptake

**Figure 7:**
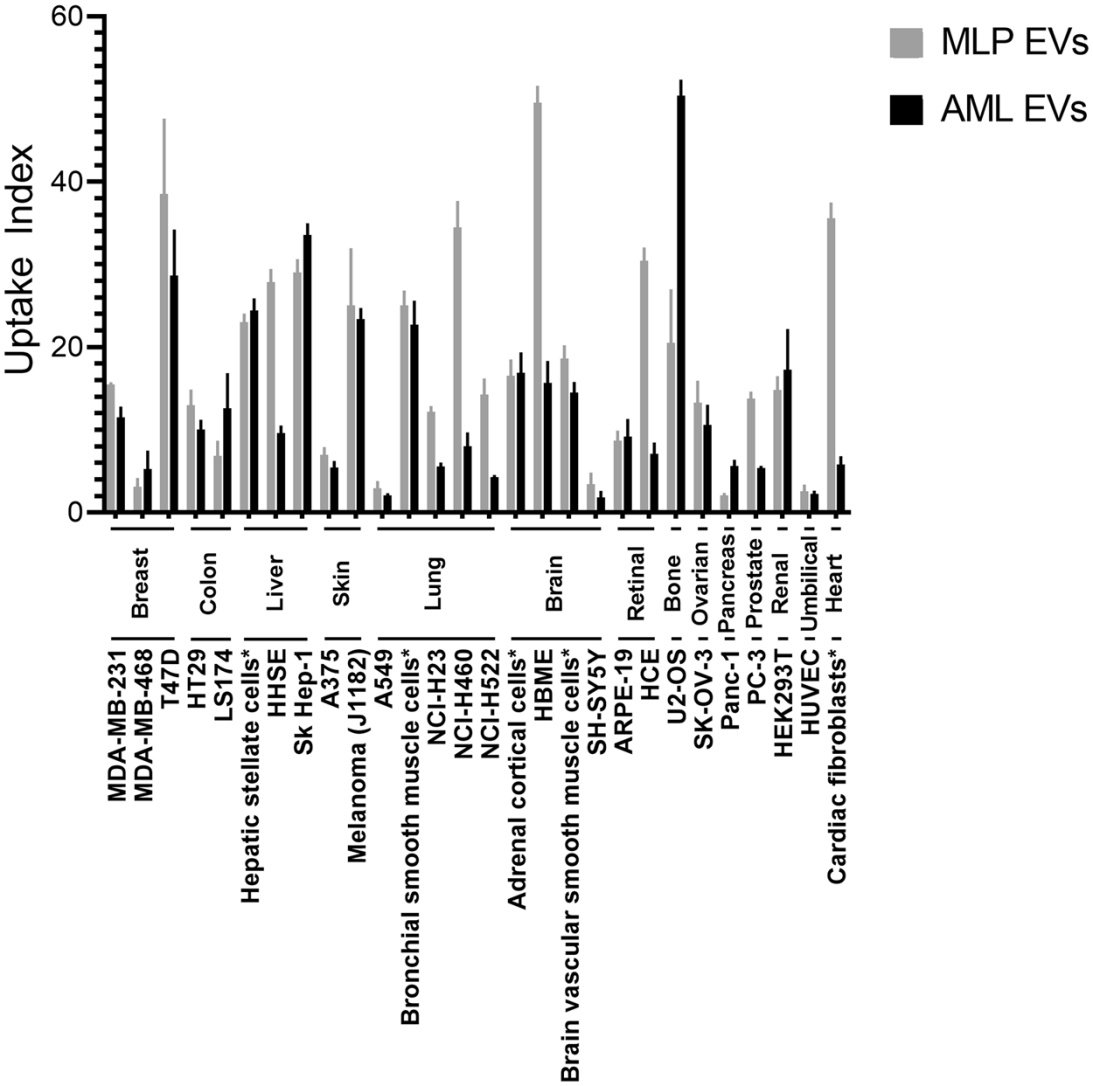
High-content uptake analysis of native EVs incubated with a panel of 28 different human cell lines. Uptake index is determined as the fold-change in fluorescence compared to a DiI background control after a 16 hour incubation, with uptake index of the DiI background thus set to ‘1’ for each cell line. Cell lines are broadly clustered by organ type and lines marked with * are primary cultures.

Considering the stark results from our flow cytometry assay, we wanted to broaden our panel of cell lines to better represent the totality of organs in the human body. As such, we have performed a high-content screening assay to assess EV uptake in a broad panel of 28 different human cell lines, including primary cultures. Comparing the uptake of native MLP29 and AML12 EVs, there was high variability in terms of the uptake capabilities of the assayed cell lines by our EV models (Fig. 7). Some lines internalised low amounts of EVs compared to others, with A549, HUVEC and SH-SY5Y amongst these. In most cases, both EVs were captured to a similar degree but some cell lines did show preference for one or another type of EV. Of the cell lines that did exhibit a preference, MLP29 EVs were uptaken to a higher degree with the largest differences seen for HBME, HCE, primary cardiac fibroblasts and the NCI lines corresponding to the lung. In contrast, U2-OS cells preferred AML12 EVs, an effect that was not evident through flow cytometry. This high uptake by a single cell line and comparatively lower uptake with others indicates the existence of a specificity encoded to the surfaces of EVs and cells that is desirable considering the application of EVs as vehicles for targeted delivery^46^. Thus, high-content uptake screening may be a useful approach for predicting biodistribution and potential off-target effects for candidate EV-encapsulated therapies. It should be noted here that EV uptake is also governed by the proteins, lipids and other molecules that comprise the vesicle surface, not just glycans. Whilst the lectin array analysis of the samples showed some differences in sialylation and fucosylation, it is likely that other surface ligands also play a role. For example, the Western blot data shows a difference in Lamp1 content between EVs from either AML12 or MLP29 which may be have an impact here. Untangling the relative contribution of these biomolecular classes to the vesicle interactome is a challenging undertaking to consider but would be useful to focus future efforts in engineered targeting of vesicles.

**Figure 8:**
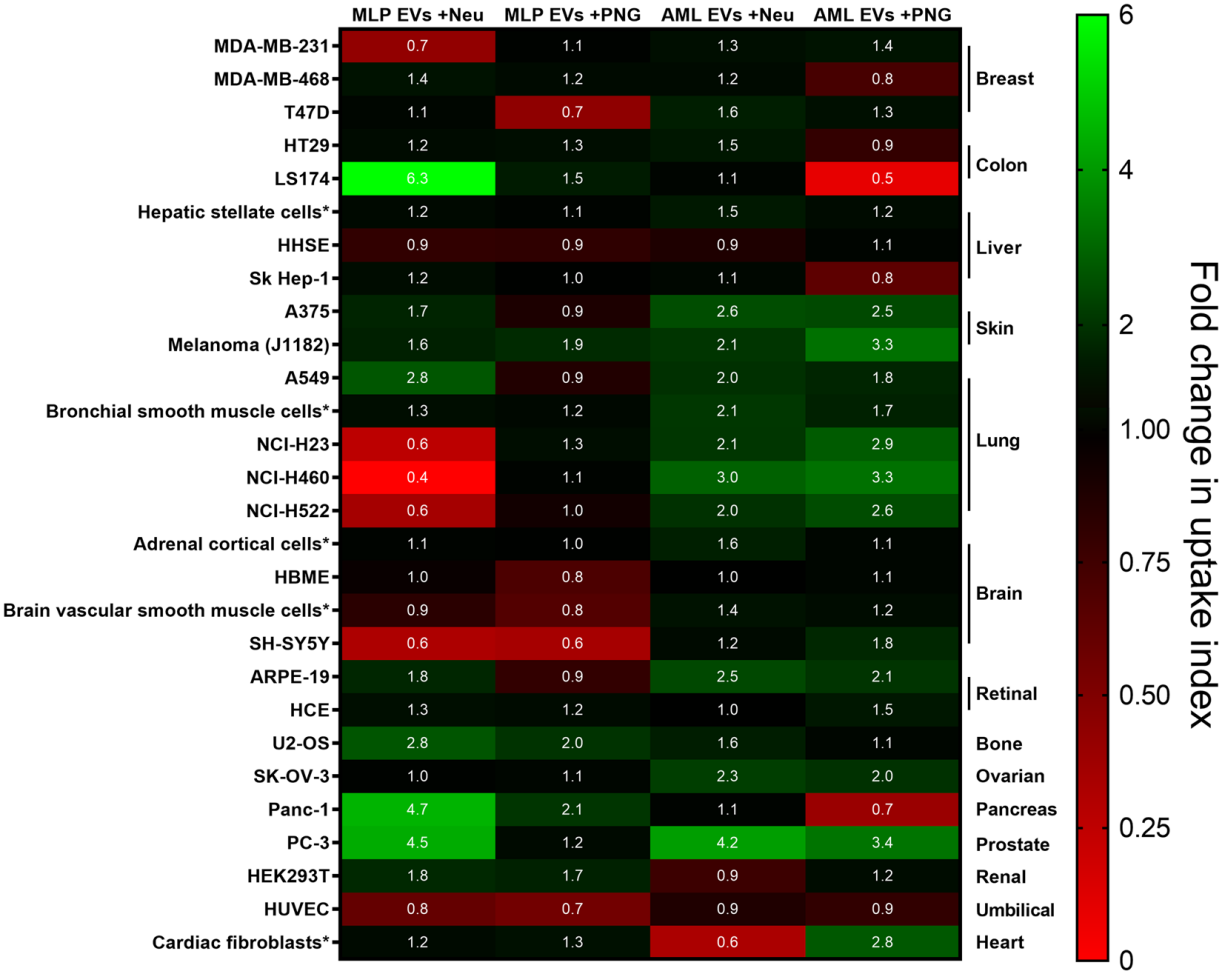
High-content uptake analysis of EVs with modified glycosylation pattern. A panel of 28 human cell lines were incubated with native EVs or with glycosidase-treated EVs during 16 hours. Confocal images were acquired and quantified as indicated in *Material and Methods* section. The heat map represents the fold change in uptake relative to the uptake of unmodified EVs.

Expanding the analysis to include EVs of modified glycosylation patterns further highlights how different cell lines behave differently towards different EVs, with no universal response to glycosylation changes (Fig. 8). Treating AML12 EVs with either of the glycosidases increases their uptake by the skin, lung, retinal, ovarian and prostate cell lines, in a reflection of our flow cytometry results. Elsewhere, glycosidase effects are more variable. Desialylation is shown to selectively depress uptake in a number of cases, particularly between MLP29 EVs and the NCI lung lines. The HUVEC and SH-SY5Y cell lines exhibited depressed uptake capabilities for MLP29 EVs after either treatment, implicating N-glycans and sialylation as important determinants in the uptake of these EVs.

Certain cell lines react differently to the effects of modifying glycosylation depending on the EV model used. LS174 is a clear example. Neuraminidase treatment of MLP29 EVs caused an increase in their uptake whereas PNGase F treatment of AML12 EVs caused a lessening. Interestingly, the breast cancer lines MDA-MB-231 and MDA-MB-468 show different responses to one another. In the case of MDA-MB-231, the only change in uptake is for MLP29 treated with neuraminidase, whereas MDA-MB-468 shows the same effect only with de-N-glycosylated AML12 EVs. Both are derived from triple-negative mammary carcinomas^47^ and their different responses are interesting in light of EV therapies against cancer^48^. Different patients would likely respond differently to the same therapy, demonstrating evidence for the value of patient stratification based on molecular characteristics of their cancers^49^.

## Discussion

An important role is emerging for glycosylation in EV biology, to include biogenesis and cargo recruitment^16^. Here, we present further evidence that the glycans of EVs are also important in their uptake by recipient cells, as disruption of native glycosylation alters this uptake in a panel of different cell lines. The current understanding of EV uptake comprises a broad range of interaction mechanisms^7,15^ and the relative contribution of these in a given system is unclear. Our data, together with the wider body of work on EV glycosylation, demonstrates glycosylation is a clear component of processes relating to EV uptake, and that disruption of native glycosylation can impinge upon the tropism of EVs towards different cell types. However, we cannot definitively state whether the observed effects are related to the initial binding of EVs to cells, subsequent internalisation processes, or both. We have demonstrated internalisation with our confocal microscopy results but these represent a snapshot after the underlying molecular events of binding and/or uptake. Further investigation of EV glycosylation in these processes is warranted, as is the expansion of the study to other EV contexts.

Herein, we used two hepatocyte-like murine cell lines for EV production due to our prior experience with these EVs, rather than an assured suitability as models for EV glycosylation studies. MLP29 derives from a spontaneously immortalised embryonic line with a clone chosen for hepatocyte markers^25^ whilst AML12 originates from a transgenic mouse with heterologously expressed TGF-α^24^ and the impact of these immortalisation routes on glycosylation is uncertain. It has been shown through flow cytometry with fluorescent lectins that cell surface glycosylation is changed upon immortalisation^50^. As such, although the native glycan content of these EVs may be altered by immortalization, in this work, we wanted to examine and explore EV glycosylation that was as close to native as possible, informing our choice of non-cancerous cell lines and our approach of using glycosidase enzymes on purified EVs coming from highly reproductible system. Other approaches to glycoengineering of cellular products act at the cellular level, either through genetic manipulation of glycosyltransferase expression^51^ or small molecule inhibition of the glycosylation machinery^52^. However, both the presence and absence of N-glycans have been implicated in the recruitment of cargo proteins during EV biogenesis^53,54^, and so it is likely that these approaches could impact upon the biomolecular content and identity of any resulting EVs. Our glycosidase approach therefore preserves the integrity of the system – perturbing EV glycosylation after the complex and varied biological processes of biogenesis. These holistic considerations extended even to our method of vesicle labelling and the use of lipophilic dyes for the functional uptake assays, instead of targeting canonical marker proteins with fluorescent antibodies or genetically tagged reporters. This latter approach necessitates overexpression of the chosen marker and may therefore bias the glycome of the system, skewing the presentation of ligands relevant to vesicle capture. Moreover, different exosome subpopulations exhibit different relative proportions of marker proteins which may further skew marker-based labelling^55^. In attempting to view the bulk EV population, nonspecific lipophilic dyes are best suited to this application, although the low fluorescence intensity of our vesicle models necessitated long incubation times of 16 hours with EVs and recipient cells in order to obtain sufficient cumulative signal. However, due to logistical constraints, we instead applied an amine-targeted label for lectin microarray results as per protocols previously developed within our group^31^ and used elsewhere in the literature^21^. We are confident that the purity of our vesicle preparations discounts interference from potentially contaminating glycoproteins, evidenced by cryo-TEM, and the high particle counts per µg of protein and strong levels of EV marker proteins.

Generally, the largest increases in EV uptake that we observed with the flow cytometry assay came from desialylation with neuraminidase. Prior work from Inder and collaborators has reported this effect of neuraminidase significantly increasing EV uptake in a model of PC3-derived exosomes supplied to RAW264.7 osteoclast precursor cells^37^. This effect was also shown for SKOV3 cells and CD9+ exosomes from this same line, although to a lesser degree^56^. Taken together with the data from our panel of cell types and EV models, this effect of desialylation appears more widely applicable. Moreover, Escrevente and colleagues demonstrated that pre-incubation of cells with any of the free monosaccharides that compose mammalian glycosylation can reduce uptake^56^, a finding supported by another study using free sialic acid^21^. We attribute these findings to the presence of specific carbohydrate-protein interactions between cell surface receptors and EV glycan motifs accessible only after trimming of terminal sialic acid residues. However, this explanation is not sufficient considering that de-N-glycosylation using PNGase F can also increase uptake. Accounting for this, we speculate that there are secondary, physical effects to modifying EV glycosylation. This is apparent from our data looking at vesicle zeta potential and the reduction of negative charge towards neutrality. Correspondingly, less electrostatic repulsion would occur between these EVs and negatively charged cell membranes and proteoglycans, resulting in increased deposition. A further possibility is that modification of glycan structures removes steric hindrance of other vesicle surface ligands that are then more able to encounter their cell surface receptors. A similar phenomenon has been recently observed with influenza virus, where a change in the glycosylation of viral haemagglutinin and neuraminidase proteins affects binding to cell surface glycans and consequently virulence^57^. These are all avenues that we aim to explore in our future research.

Our high-content uptake analysis makes clear that EVs from nominally similar cell lines such as MLP29 and AML12 can have starkly different propensities for uptake depending on the receiving cells. However, screening a panel of cell lines *in vitro* is not sufficient to replace *in vivo* studies, as evidenced by a recent publication^23^. Therein, MLP29 EVs treated with neuraminidase exhibited a different biodistribution when injected into mice, with an increase in lung accumulation. Our data with lung cell lines showed an opposite effect, with uptake lessened, and it is unclear whether this is a species-specific effect or due to other factors that cannot be reconstituted in plate-based assays e.g. lymphatic systems. Perhaps the clearest conclusion from the screening is that the landscape of vesicle-cell interactions is huge. Herein we have targeted only a sub-section of possible glycan structures and have already seen widely varying results. This neatly demonstrates the challenges and complexity of EV studies as idiographic – that any conclusions drawn may only be valid with that specific type of EV with the specific cell lines used. This reflects also a concept in our work, whereby we have assayed the uptake of murine EVs by human cell lines. Whilst protein ligands are likely to show species-specific sequences, mammalian glycans are more universal. We believe that the cross-species approach that we have used may better show the impact of modifying EV glycosylation.

This work is potentially impactful in light of current efforts towards the development of EV therapeutics^48,58–60^. Importantly, scale up of EV production to meet clinical demands may introduce variation into the glycosylation of EV products and could therefore impact upon product efficacy^16^, a hypothesis strengthened by our findings herein. To combat the possibility of affecting EV products during development, we recommend that therapy developers look to incorporate glycan characterisation as a means of quality control throughout product development.

## Supplementary information

Supplementary Information

Supplementary Datasets S2 and S4

## Data Availability

The datasets generated during and/or analysed during the current study are available from the corresponding authors on reasonable request.
