# Supplementary material for: Assessing the role of surface glycans of extracellular vesicles on cellular uptake
Source: Sci Rep. 2019 Aug 15;9:11920. doi: 10.1038/s41598-019-48499-1 (PMC6695415; doi:10.1038/s41598-019-48499-1)

Assessing the role of surface glycans of extracellular vesicles on cellular uptake – Supplementary Information

# Authors:

# Charles Williams^1,2^, Raquel Pazos^2^, Félix Royo^1,3^, Esperanza González^1^, Meritxell Roura-Ferrer^4^, Aitor Martinez^4^, Jorge Gamiz^4^, Niels‑Christian Reichardt^2,5^ and Juan M Falcón-Pérez^1,3,4^

Supplementary Table 1, Lectins employed in the study, suppliers and binding specificities.

| **Lectin** | **Origin** | **Supplier** | **Specificity** | **Broad saccharide preference** |
| --- | --- | --- | --- | --- |
| ABL | *Agaricus bisporus* | Sigma-Aldrich | β-Gal(1-3)GalNAc, T antigen | T antigen |
| ACA | *Amarantus caudatus* | Vector Labs | β-Gal(1-3)GalNAc, T antigen | T antigen |
| AHA | *Arachis hypogaea* | Sigma-Aldrich | β-Gal(1-3)GalNAc, T antigen | GalNAc |
| AOL | *Aspergillus orzyae* | TCI Chemicals | Fuc | Fuc |
| ASA | *Allium sativum* | EY Labs | α-1,3-Man | Man |
| BS-I (EY) | *Griffonia simplicifolia* I | EY Labs | αGal | αGal |
| BSI-B4 | *Griffonia simplicifolia* isolectin B4 | Vector Labs | αGal | αGal |
| BS-II | *Griffonia simplicifolia* II | Vector Labs | Terminal GlcNAc | GlcNAc |
| CAL | *Cicer arietinum* | EY Labs | GalNAc, Lac | LacNAc |
| ConA | Concanavalin A | Vector Labs | α-Man, α-Glc | Man |
| DBL | *Dolichos biflorus* | Sigma-Aldrich | α-GalNAc | GalNAc |
| DSL | *Datura stramonium* | Sigma-Aldrich | (GlcNAc)2, LacNAc | GalNAc |
| ECA | *Erythrina crista-galli* A | Vector Labs | Gal, GalNac | Gal |
| EEA | *Euonymus europaeus* | Vector Labs | Lac, blood groups B and H | αGal |
| GS-I | *Griffonia simplicifolia* I | Vector Labs | α-Gal | αGal |
| HHA | *Hippeastrum* hybrid | Vector Labs | α-1,3-Man, α-1,6-Man | Man |
| HMA | *Homarus americanus* | EY Labs | Sia,GalNAc | Sia |
| HPL | *Helix pomatia* | Sigma-Aldrich | Terminal α-GalNAc | GalNAc |
| JAC | *Artocarpus integrifolia* | Sigma-Aldrich | GalNac, T antigen | T antigen |
| LCA | *Lens culinaris* | Vector Labs | Complex Man/GlcNAc core with α‑1,6Fuc | Fuc |
| LEL | *Lycopersicon esculentum* | Sigma-Aldrich | (GlcNAc)_3_ | GlcNAc |
| LTL | *Lotus tetragonolobus* | Vector Labs | Terminal α-Fuc, sialyl-Lewis X | Fuc |
| MAL-I | *Maackia amurensis* lectin I | Vector Labs | α-2,3-Sia, LacNac | Sia |
| MAL-II | *Maackia amurensis* lectin II | Vector Labs | LacNAc | Gal |
| MOA | *Marasmium oreades* agglutinin | EY Labs | Gal-α-1,3-Gal, Gal-α-1,3-Gal-β-1,4-GlcNAc | αGal |
| MPA | *Maclura pomifera* | Vector Labs | T antigen, αGalNAc | T antigen |
| NPL | *Narcissus pseudonarcissus* | Vector Labs | Terminal Man, Man | Man |
| PAL | *Pseudomonas aeruginosa* PA-I | Sigma-Aldrich | Gal, GlcNAc | GlcNAc |
| PHA | *Phaseolus vulgaris* agglutinin E+L | Vector Labs | Complex branched chain glycans | LacNAc |
| PHA L | *Phaseolus vulgaris* agglutinin | Vector Labs | Complex branched chain glycans | LacNAc |
| PNA | Peanut agglutinin | Vector Labs | T antigen, α-1,3-Gal, GalNAc | GalNAc |
| PSA | *Pisum sativum* | Vector Labs | Fuc, α-1,6-GlcNAc, α-Man | Fuc |
| PT-I | *Psophocarpus tetragonolobus* I | Vector Labs | αGalNAc | GalNAc |
| PT-II | *Psophocarpus tetragonolobus* II | Vector Labs | αGal, α-1,2-Fucosylated LacNAc | αGal |
| PWA | *Phytolacca americana* | Sigma-Aldrich | (GlcNAc)_3_ | GlcNAc |
| RCA | *Ricinus communis* agglutinin, | Vector Labs | β-Gal, Lac, LacNAc | Gal |
| SBAmax | Soybean agglutinin | Vector Labs | αGal-GalNAc | GalNAc |
| SJA | *Sophora japonica* | Vector Labs | GalNAc | GalNAc |
| SNA | *Sambucus nigra* | Vector Labs | α-2-6-Sialylated LacNAc | Sia |
| SSA | *Salvia sclarea* | EY Labs | Terminal GalNAc-serine | GalNAc |
| STL | *Solanum tuberosum* | Vector Labs | (GlcNAc)_3_, LacNAc | GlcNAc |
| UEA-I | *Ulex europaea* agglutinin | Vector Labs | α-1,3-Fuc, L-Fuc | Fuc |
| VFA | *Vicia faba* | EY Labs | α-Man, Glc, GlcNAc | Man |
| VVL | *Vicia villosa* | Vector Labs | T antigen, GalNAc | T antigen |
| WFL | *Wisteria floribunda* | Vector Labs | GalNAc | GalNAc |
| WGA | *Triticum vulgaris* | Vector Labs | (GlcNAc)_n_, Sia | GlcNAc |

Supplementary Table S3, Human cell lines assayed in high-content uptake experiments

| **Cell Line** | **Representative Organ** | **Source** | **Media** | **Coating** |
| --- | --- | --- | --- | --- |
| MDA-MB-231 | Breast | ATCC® HTB-26™ | RPMI supplemented with 10%FBS and NEAA | N/A |
| MDA-MB-468 | Breast | ATCC® HTB-132™ | RPMI supplemented with 10%FBS and NEAA | N/A |
| T47D | Breast | ATCC® HTB-133™ | RPMI supplemented with 10%FBS and NEAA | N/A |
| HT29 | Colon | ATCC® HTB-38™ | McCoy supplemented with 10%FBS and NEAA | N/A |
| LS174 | Colon | ATCC® CL-188™ | DMEM supplemented with 10%FBS and NEAA | N/A |
| Hepatic stellate cells | Liver | Primary line, Innoprot proprietary | Innoprot proprietary media with 2% FBS | Collagen type I |
| HHSE (Human Hepatic Sinusoidal Endothelial cells) | Liver | Immortalised primary line, Innoprot proprietary | Innoprot proprietary media with 5% FBS | Collagen type I |
| Sk Hep-1 | Liver | ATCC® HTB-52™ | RPMI supplemented with 10%FBS and NEAA | N/A |
| A375 | Skin | ATCC® CRL-1619™ | RPMI supplemented with 10%FBS and NEAA | N/A |
| Melanoma (J1182) | Skin | Primary line, Innoprot proprietary | RPMI supplemented with 10%FBS and NEAA | N/A |
| A549 | Lung | ATCC® CCL-185™ | F-12K supplemented with 10%FBS and NEAA | N/A |
| Bronchial smooth muscle cells | Lung | Primary line, Innoprot proprietary | Innoprot proprietary media with 2% FBS | Collagen type I |
| NCI-H23 | Lung | ATCC® CRL-5800™ | RPMI supplemented with 10%FBS and NEAA | N/A |
| NCI-H460 | Lung | ATCC® HTB-177™ | RPMI supplemented with 10%FBS and NEAA | N/A |
| NCI-H522 | Lung | ATCC® CRL-5810™ | RPMI supplemented with 10%FBS and NEAA | N/A |
| Adrenal cortical cells | Neuronal | Primary line, Innoprot proprietary | Innoprot proprietary media with 5% FBS | Collagen type I |
| HBME (Human Brain Microvascular Endothelial cells) | Neuronal | Immortalised primary line, Innoprot proprietary | Innoprot proprietary media with 5% FBS | Collagen type I |
| Brain vascular smooth muscle cells | Neuronal | Primary line, Innoprot proprietary | Innoprot proprietary media with 2% FBS | Collagen type I |
| SH-SY5Y | Neuronal | ATCC® CRL-2266™ | RPMI supplemented with 10%FBS and NEAA | N/A |
| ARPE-19 | Retinal | ATCC® CRL-2302™ | DMEM/F12 supplemented with 10%FBS and NEAA | N/A |
| HCE (Human Conjunctival Epithelial cells) | Retinal | Immortalised primary line, Innoprot proprietary | Innoprot proprietary media with 2% FBS | Collagen type I |
| U2-OS | Bone | ATCC® HTB-96™ | DMEM/F12 supplemented with 10%FBS and NEAA | N/A |
| SK-OV-3 | Ovarian | ATCC® HTB-77™ | McCoy supplemented with 10%FBS and NEAA | N/A |
| Panc-1 | Pancreas | ATCC® CRL-1469™ | DMEM supplemented with 10%FBS and NEAA | N/A |
| PC-3 | Prostate | ATCC® CRL-1435™ | F-12K supplemented with 10%FBS and NEAA | N/A |
| HEK293T | Renal | ATCC® CRL-3216™ | DMEM supplemented with 10%FBS and NEAA | N/A |
| HUVEC | Umbilical | Primary line, Innoprot proprietary | Innoprot proprietary media with 5% FBS | Collagen type I |
| Cardiac fibroblasts | Heart | Primary line, Innoprot proprietary | Innoprot proprietary media with 5% FBS | Collagen type I |

# antiLamp1 Western Blot


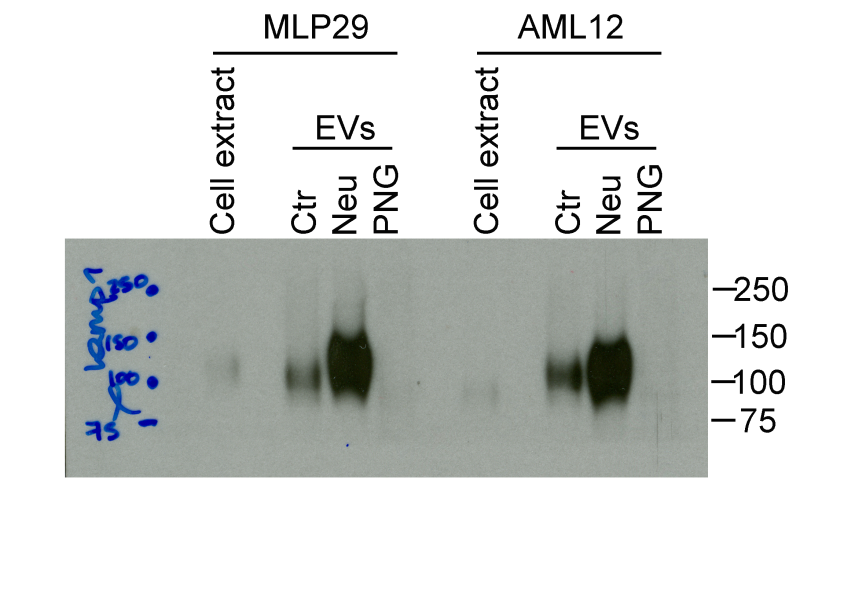


# antiLimpII Western Blot


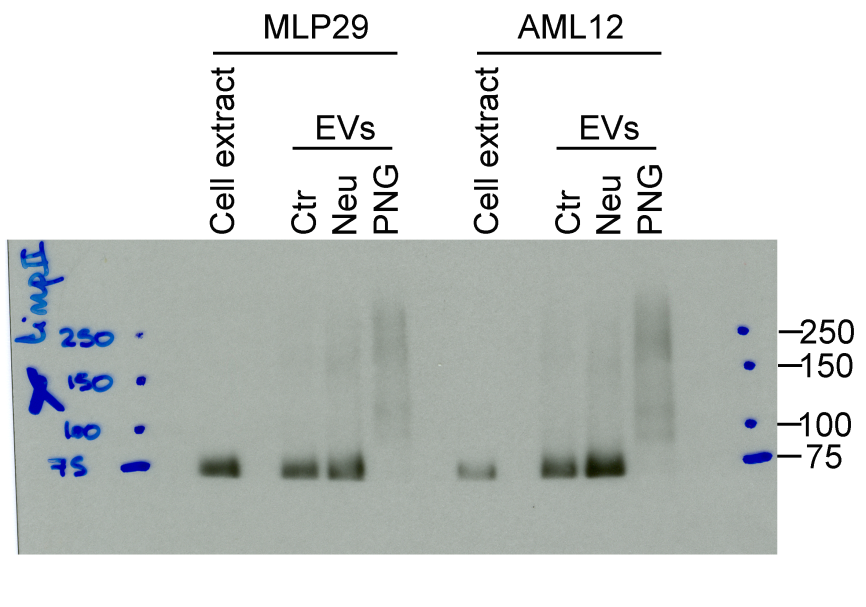


# antiTSG101 Western Blot


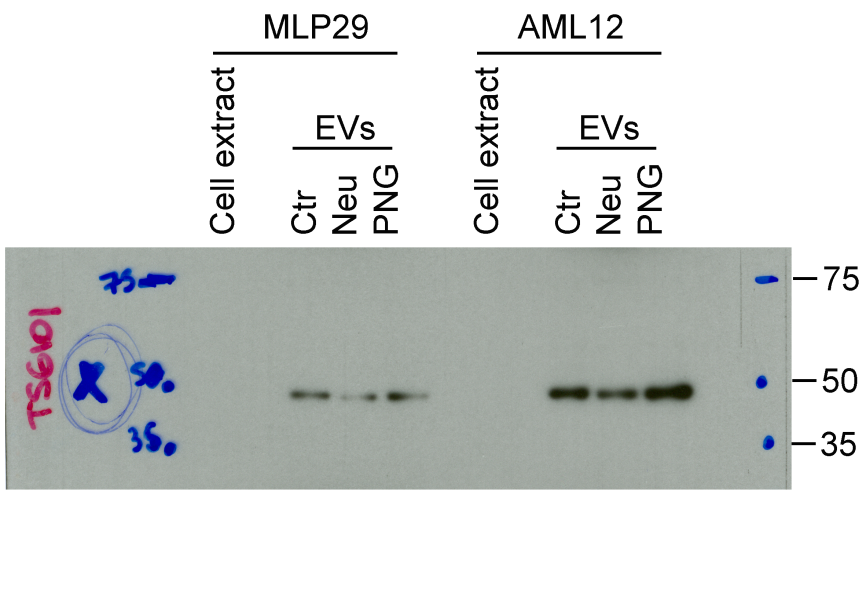


# antiHsp70 Western Blot


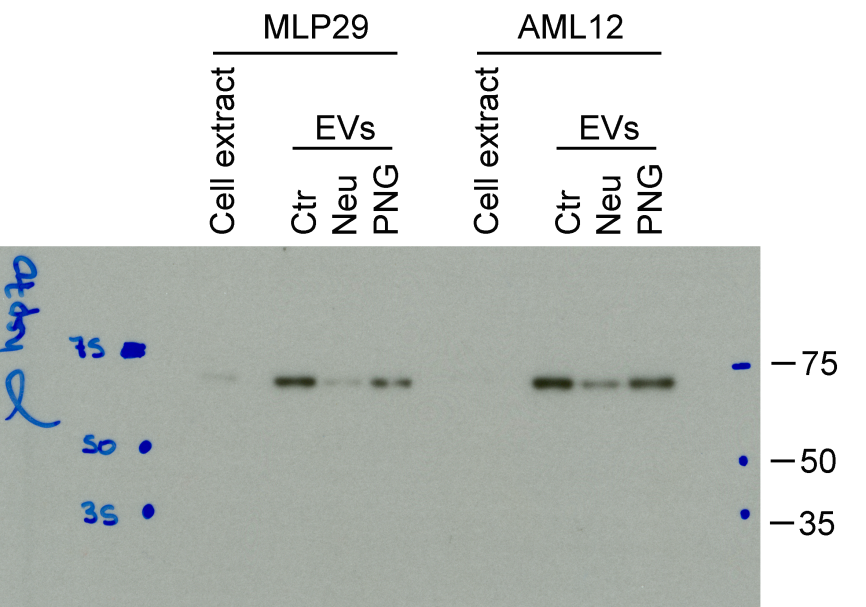


# antiGrp78 Western Blot


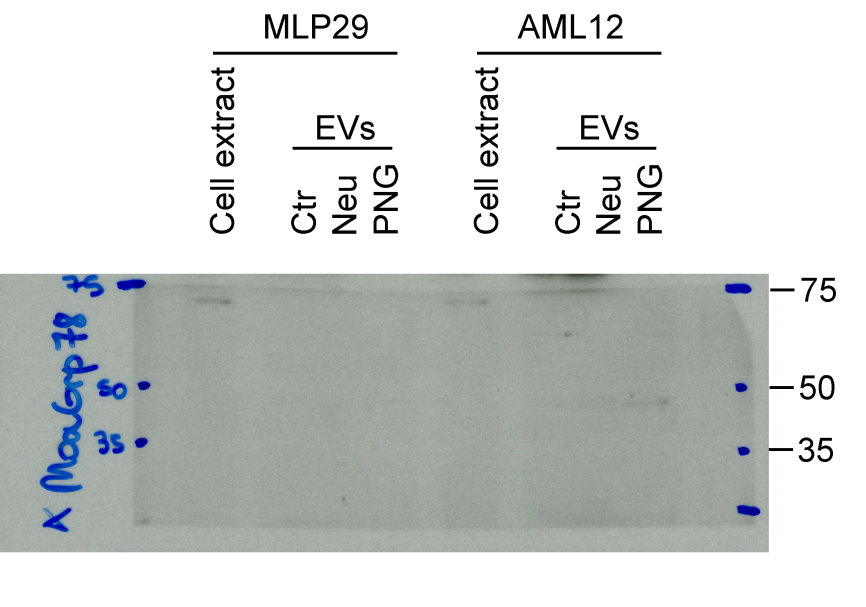


# Flow cytometry gating example


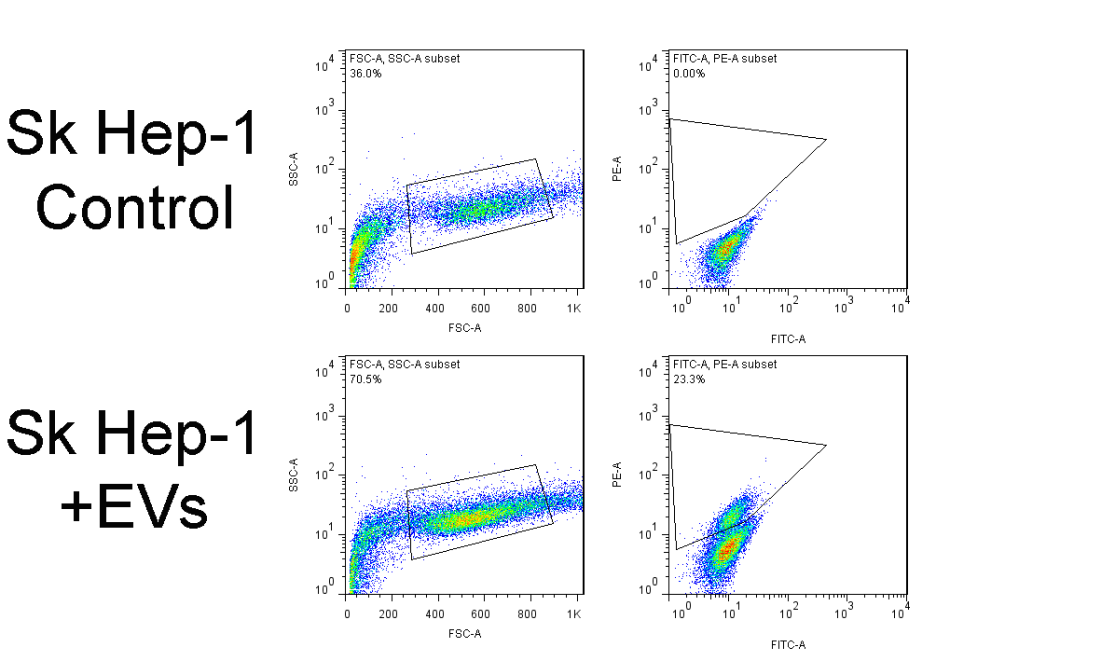

Supplement: Supplementary file 1 — Supplementary Information [file 41598_2019_48499_MOESM1_ESM.docx]
